# Supplementary material for: Seminoma and Embryonal Carcinoma Footprints Identified by Analysis of Integrated Genome-Wide Epigenetic and Expression Profiles of Germ Cell Cancer Cell Lines
Source: PLoS One. 2014 Jun 2;9(6):e98330. doi: 10.1371/journal.pone.0098330 (PMC4041891; doi:10.1371/journal.pone.0098330)
Supplement: File S1 — ZIP file containing DMRforPairs output for significant regions. Please start from the html files. (ZIP) [file pone.0098330.s008.zip › figures/39489.pdf]

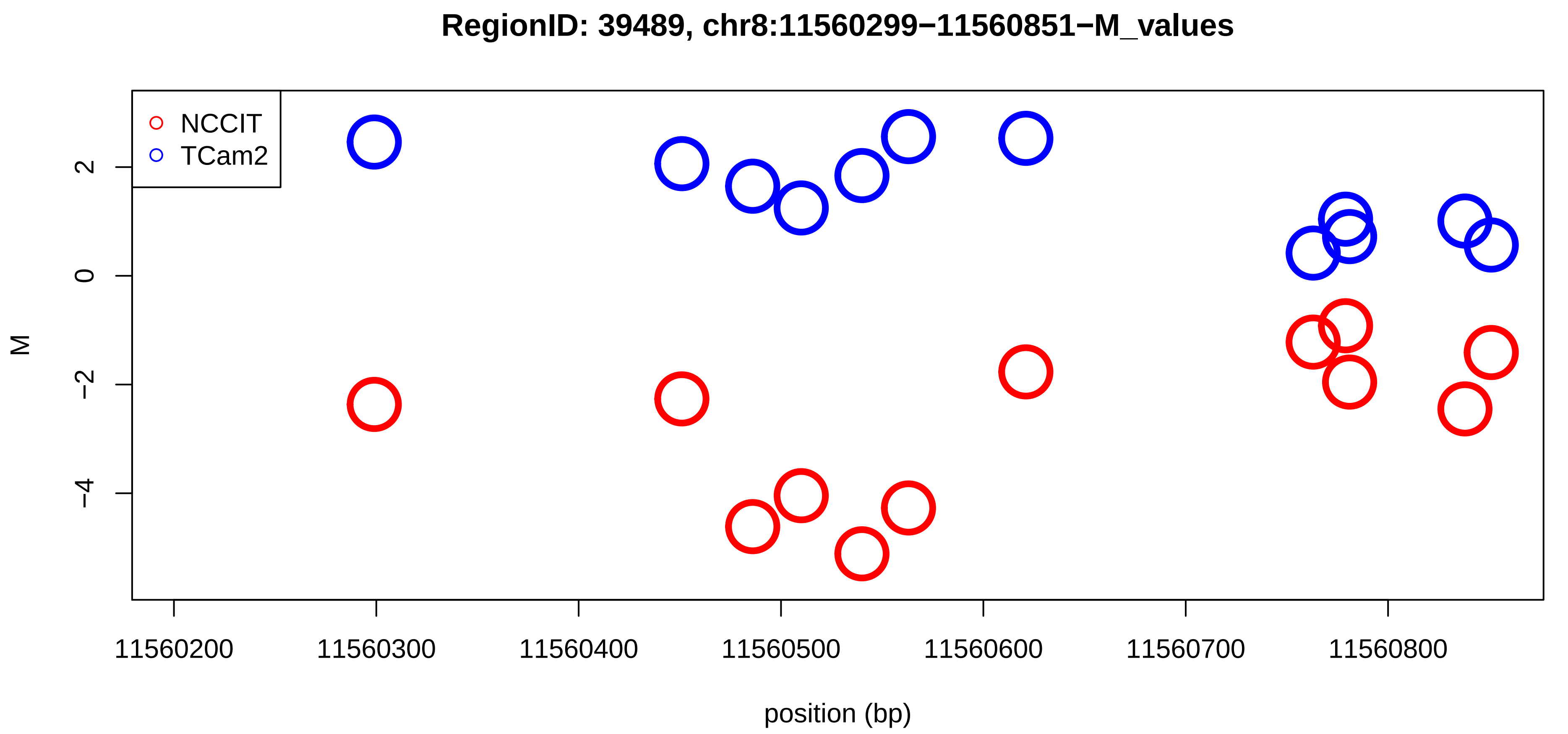
RegionID: 39489, chr8:11560299−11560851−M_values
NCCIT
TCam2
M
2
0
−2
−4
11560200
11560300
11560400
11560500
11560600
11560700
11560800
position (bp)

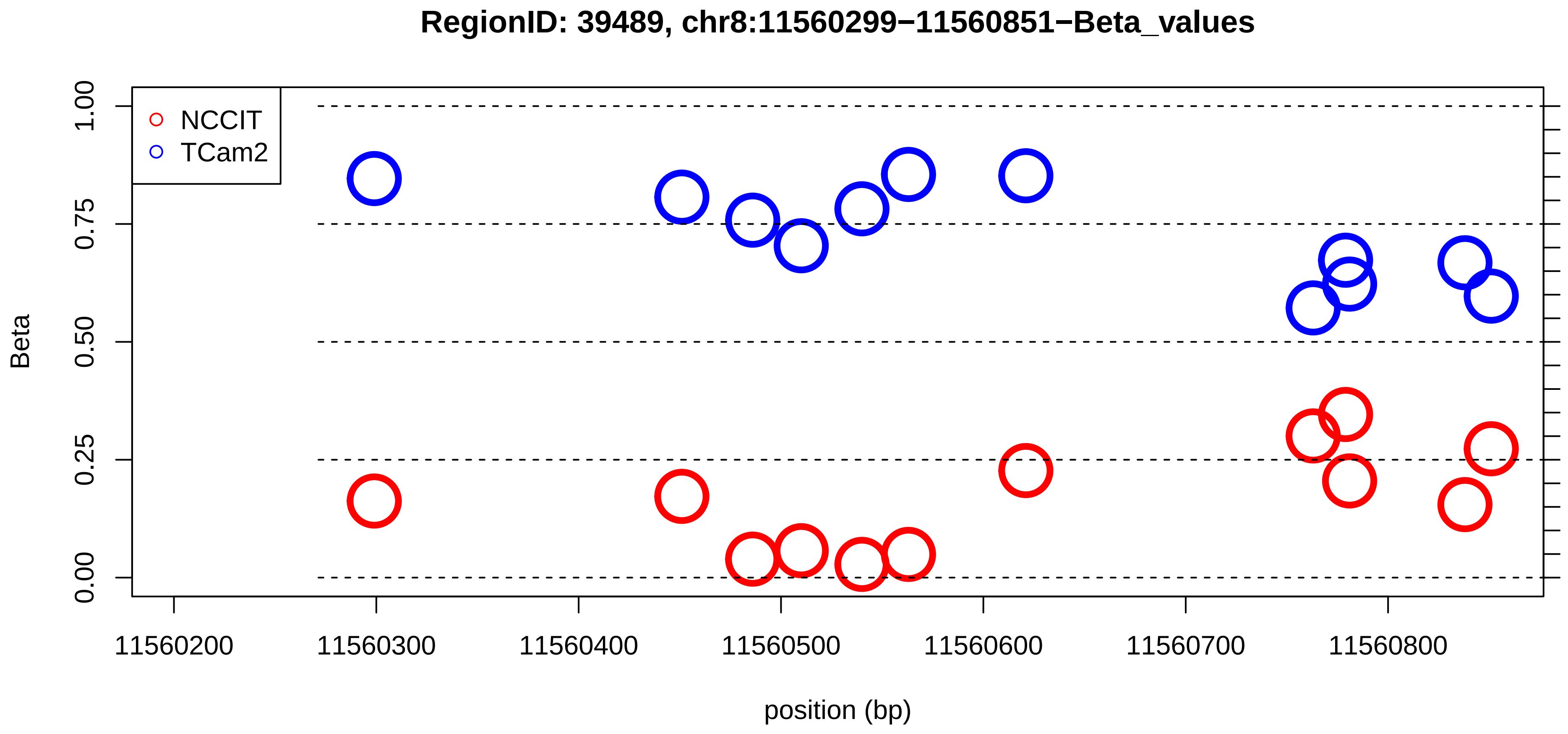
RegionID: 39489, chr8:11560299−11560851−Beta_values
NCCIT
TCam2
Beta
1.00
0.75
0.50
0.25
0.00
11560200
11560300
11560400
11560500
11560600
11560700
11560800
position (bp)

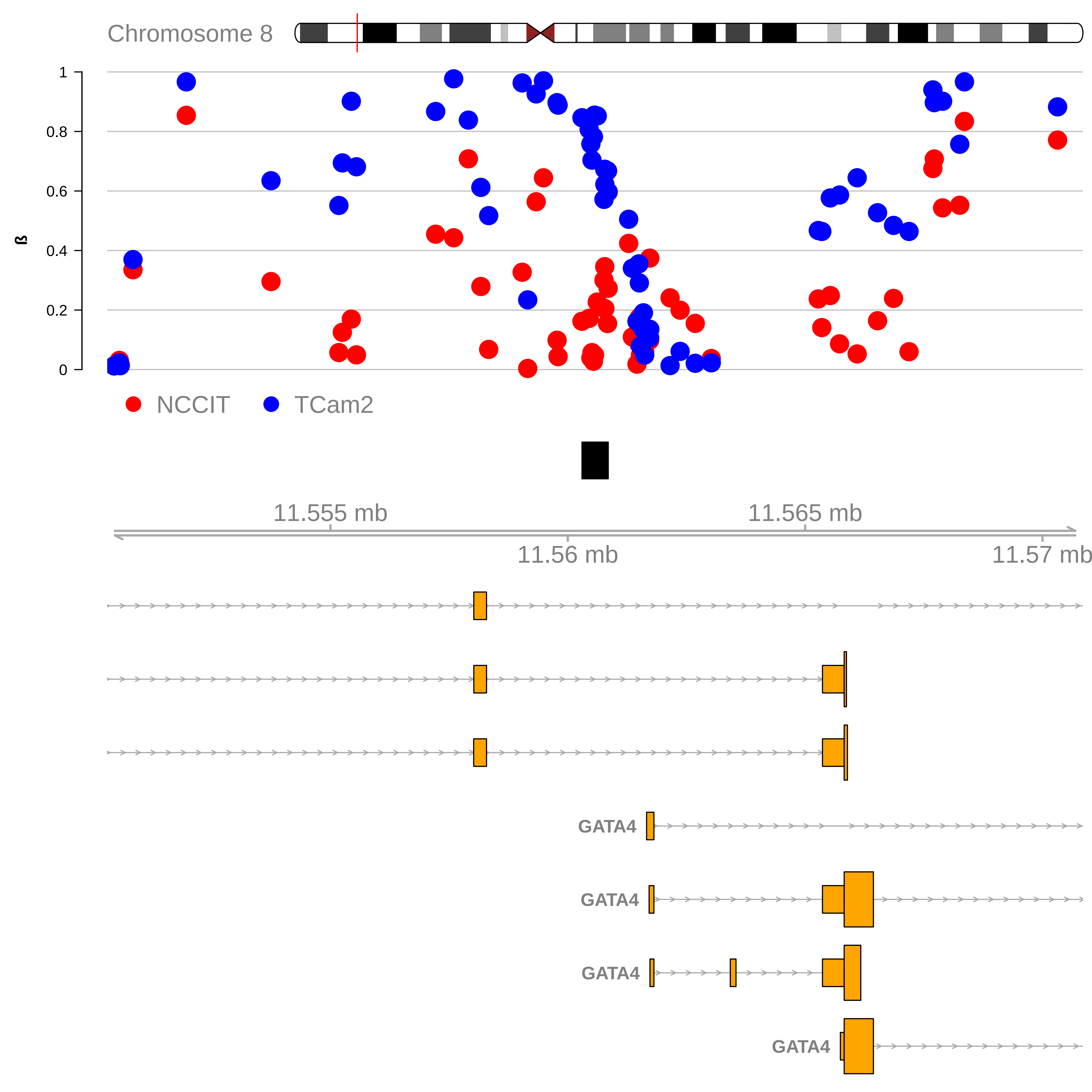

Chromosome 8
1
0.8
0.6
0.4
0.2
0
ß
NCCIT
TCam2
11.555 mb
11.565 mb
11.56 mb
11.57 mb
GATA4
GATA4
GATA4
GATA4
